# One-Carbon Metabolism Links Nutrition Intake to Embryonic Development via Epigenetic Mechanisms

**DOI:** 10.1155/2019/3894101

**Published:** 2019-03-10

**Authors:** Si Wu, Jun Zhang, Feifei Li, Wei Du, Xin Zhou, Mian Wan, Yi Fan, Xin Xu, Xuedong Zhou, Liwei Zheng, Yachuan Zhou

**Affiliations:** ^1^State Key Laboratory of Oral Diseases, National Clinical Research Center for Oral Diseases, Department of Pediatric Dentistry, West China Hospital of Stomatology, Sichuan University, Chengdu, Sichuan 610041, China; ^2^State Key Laboratory of Oral Diseases, National Clinical Research Center for Oral Diseases, Department of Cariology and Endodontics, West China Hospital of Stomatology, Sichuan University, Chengdu, Sichuan 610041, China

## Abstract

Beyond energy production, nutrient metabolism plays a crucial role in stem cell lineage determination. Changes in metabolism based on nutrient availability and dietary habits impact stem cell identity. Evidence suggests a strong link between metabolism and epigenetic mechanisms occurring during embryonic development and later life of offspring. Metabolism regulates epigenetic mechanisms such as modifications of DNA, histones, and microRNAs. In turn, these epigenetic mechanisms regulate metabolic pathways to modify the metabolome. One-carbon metabolism (OCM) is a crucial metabolic process involving transfer of the methyl groups leading to regulation of multiple cellular activities. OCM cycles and its related micronutrients are ubiquitously present in stem cells and feed into the epigenetic mechanisms. In this review, we briefly introduce the OCM process and involved micronutrients and discuss OCM-associated epigenetic modifications, including DNA methylation, histone modification, and microRNAs. We further consider the underlying OCM-mediated link between nutrition and epigenetic modifications in embryonic development.

## 1. Introduction

Nutrition encompasses the relationships between development and a multitude of processes such as ingestion and digestion of food for metabolism and synthesis of nutrients and is profoundly influenced by various lifestyle factors and eating habits [1]. Different dietary factors like carbohydrates, proteins, lipids, and microelements are all “fundamental materials” for organism development. These nutrient substances and their metabolites not only supply adequate energy for cell activities but also play regulatory roles in various pathways of basal metabolism [2]. Pregnancy is a critical period of cell division and differentiation occurring in utero. The maternal nutritional status greatly influences the fetal development, pregnancy outcome, and further disease development of offspring [3–6]. In the early stages of fetal development, the stem cell fate determination is regulated by epigenetic modification, which is closely related with the metabolic supply from maternal nutrition intake [7]. The remarkable breakthroughs in exploring epigenetic mechanisms have coincided with the focus on the roles of diet and nutrient metabolites in fetal development [8]. Several recent studies reported a potential interplay between gene expression and metabolic microenvironment, which is involved in modulating and regulating the epigenome of cells during early development and stem cell fate determination [9].

The one-carbon metabolism (OCM) is a vital metabolic process involved in the methyl group donation or transfer during cellular activities. These metabolic pathways utilizing one-carbon unit and related micronutrients provide essential signals involved in the interplay between biochemical pathways and epigenetic mechanisms. In this review, we summarize recent studies on the interaction between epigenetics and nutrition underlying one-carbon metabolism, including their roles in early life development and stem cell fate determination. We also highlight the identification of potential molecular targets, with an update on modulating cell fate as a therapeutic strategy.

## 2. One-Carbon Metabolism and Related Micronutrients

### 2.1. One-Carbon Metabolism (OCM)

**Figure 1:**
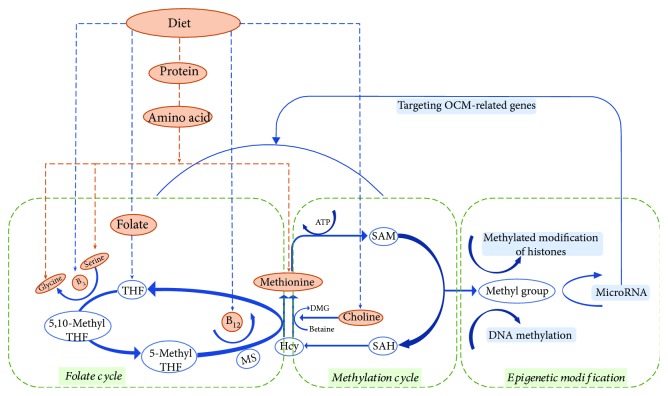
The interplay between one-carbon metabolism (OCM) and epigenetic modifications. Methionine and folate cycles are entwined and contribute to the methyl group transfers that may cause epigenetic changes in cells. Methionine is initially converted into SAM, and then, SAM actively contributes the methyl group to DNA, proteins, and other metabolites, via reactions catalyzed by substrate-specific methyltransferases. SAH, a byproduct generated from the methylation cycles, is subsequently reversibly cleaved into Hcy. Methionine can be regenerated via the process of folate cycle, which involves remethylation of Hcy by 5-methyl-THF to form methionine in the presence of vitamin B_12_ as a cofactor. SAM: S-adenosyl methionine; SAH: S-adenosyl homocysteine; Hcy: homocysteine; THF: tetrahydrofolic acid; 5,10-methyl-THF: 5,10-methylate tetrahydrofolic acid; 5-methyl-THF: 5-methylate tetrahydrofolic acid; B_12_: vitamin B_12_; B_2_: vitamin B_2_; MS: methionine synthase; DMG: dimethylglycine.

During the process of embryogenesis, metabolites and associated biochemical pathways are essential for cellular activity and stem cell fate determination. Among these metabolic processes, OCM is widely studied for the effect of one-carbon addition, transfer, or removal on cellular activity [10]. OCM is a cyclical network that includes a series of processes such as folate and methionine cycles, nucleotide synthesis, and methyl transferase reactions (Figure 1). Various metabolites in these cycles participate in the methyl (one-carbon units) group transfer and are subsequently involved in major epigenetic and epigenomic mechanisms.

Methionine and folate cycles are entwined and contribute to the methyl group transfers in key methylation reactions that may cause epigenetic changes in cells. Under an ATP-driven reaction, methionine, the immediate source of the methyl groups, is initially converted into S-adenosyl methionine (SAM) by methionine adenosyl transferase (MAT) [11]. SAM then actively contributes the methyl group to DNA, proteins, and other metabolites, via reactions catalyzed by substrate-specific methyltransferases [12]. The S-adenosyl homocysteine (SAH), a byproduct generated from the methylation cycles, is subsequently reversibly cleaved into homocysteine (Hcy) [13, 14]. During these cycles, the released methyl groups become an essential signal participating in cellular methyltransferase reactions feeding into epigenetic mechanisms. Generally, cellular methyltransferases show a higher affinity of binding SAH than SAM. Thus, almost all the SAM-dependent methylation reactions rely on SAH removal [13]. Methionine can be regenerated via the process of folate cycle, which involves remethylation of Hcy by 5-methyltetrahydrofolic acid (5-methyl-THF) to form methionine in the presence of vitamin B_12_ as a cofactor [13]. Notably, 5-methyl-THF is a one-carbon donor playing a role in the methyl group transfers underlying the process of amino acid and vitamin metabolism.

### 2.2. OCM-Related Micronutrients

Methionine is an essential amino acid and primary methyl donor in the methylation cycle of OCM. Notably, methionine metabolism can be influenced by nutritional deficiencies of relevant cosubstrates and coenzymes derived from vitamin B complex and abnormalities in their metabolism [13]. Vitamin B family consists of eight compounds, which function as coenzymes in synergistic reactions. Among these, vitamin B_9_ (folate) is the most studied owing to its crucial role in cellular metabolism during embryonic development. Folate in OCM acts as a coenzyme in the formation of tetrahydrofolate (THF), which is involved in the methyl group transfers. Vitamins B_6_ (pyridoxine) and B_12_ (cobalamin) are also indispensable for their functions in the folate cycle as cofactors in OCM. B_12_, as mentioned above, plays as a cofactor during regeneration of methionine, while B_6_ is essential for the transfer of sulfur (thiol) in the transsulfuration pathway of Hcy [15]. Timely and optimal supplementation of vitamin B from food and dietary supplements during the periconceptional period is known to promote neural tube development and protect against birth defects of offsprings [16].

Choline and betaine are important metabolites widely existing in mammals and plants. Under conditions of folate deficiency, choline and betaine provide the methyl groups and catalyze the Hcy conversion into methionine in an alternative pathway [17]. Since the concentrations of choline and betaine were found to be higher in the umbilical cord than in the maternal plasma, they are likely required for fetal development [18]. Moreover, studies with animal models suggested that maternal choline deficiency or supplementation has effects on neuron development during the second trimester of gestation and later development of offspring [19, 20].

The status of folate, cobalamin, choline, and betaine and their interactions during pregnancy have direct effects on OCM and subsequently regulate fetal growth and pregnancy outcome [21]. OCM with its related nutrient substances is ubiquitously present in stem cells during early stage of fetal development. The maternal dietary intake influences the key metabolic reactions in OCM and potentially participates in subsequent DNA synthesis and epigenetic modification via methylation reactions. As a result, OCM influences gene expression and cellular functions such as proliferation, metabolism, pluripotency, and cytodifferentiation and may regulate the growth of the embryo and fetus and even affect future disease development in offsprings.

## 3. Mechanisms of Epigenetic Modification

Epigenetics involves the study of changes in gene expression without any fundamental alterations in the DNA sequence. The genome can be functionally modified at several levels of regulation without changing the nucleotide sequence that is genetically inherited [22]. The complex epigenetic alterations include DNA methylation, histone modifications, chromatin remodeling, and noncoding RNA (ncRNA) regulation [23, 24]. These epigenetic modifications converge to modulate chromatin structure and transcription programs, allowing or preventing the access of the transcriptional machinery to genomic information [25]. Thus, the expression of gene sequences can be “switched on or off” for timely gene activation or repression during cell lineage determination. Various studies have revealed that the epigenome profiles differed in specific cell types and differentiation stages.

### 3.1. DNA Methylation

DNA methylation describes a process wherein the methyl groups are added to DNA molecules, like cytosine and adenine. The methylation process does not change the DNA sequence but may affect the activity of a DNA segment. The methylation status of a DNA sequence regulates gene expression by modulating the chromatin structure and consequently regulates the development and maintenance of cellular homeostasis [25, 26]. The pattern of DNA methylation in mammals is mostly erased and then reestablished between generations, with the demethylation and remethylation processes occurring each time during early embryogenesis [27]. It should be noted that the DNA methylation at individual genomic regions is a dynamic pattern influenced by nutritional, environmental, and other factors [26, 28]. A family of DNA methyltransferases (DNMTs) catalyzes these methylation reactions [29]. DNMTs, associated with the methylation cycle of OCM, attach the methyl groups to the carbon-5 position of cytosine, resulting in the generation of 5-methylcytosine. These epigenetic processes occur during specific stages of organism development and dynamically change during the lifespan [30].

### 3.2. Histone Modification

Nucleosomes, the basic structural units of chromatin, are formed by DNA sequences wrapped around histone proteins (H2A, H2B, H3, and H4). The amino-terminal tails of histones can be biochemically modified in multiple ways, including methylation, phosphorylation, acetylation, and ubiquitination [31]. Posttranslational modifications of histone proteins result in distinct landscapes in the cellular epigenome and determine the cell lineage fate by regulating transcriptional and metabolic activities [32]. Studies have uncovered that the histone modification patterns can be diagnostic for the cell type and differentiation stage in the embryos and embryonic stem cells [30]. Among these modifications, methylation of histones can modulate gene transcription depending on how many methyl groups are attached and which amino acids are in the methylated histones. Histone methylation status is mediated by the histone methyltransferase and demethylases, which donate or transfer the methyl groups as part of OCM. These histone-modifying enzymes are modulated by maternal dietary habits and nutritional intake and are linked to the early development of offspring as discussed below [33].

### 3.3. MicroRNA

Noncoding RNA (ncRNA) is a group of regulatory RNAs that do not code for a protein, but rather function to regulate gene expression at multiple regulatory levels, thereby influencing cellular physiology and development [34, 35]. NcRNAs include long noncoding RNA (lncRNA), microRNA (miRNA), and small interfering RNA (siRNA). Among these, miRNA is widely studied for its function in various cellular activities including proliferation, differentiation, and apoptosis. miRNA is a category of short (~21 nucleotides) ncRNAs that affect gene expression in a posttranscriptional mechanism, wherein the miRNA directly binds to the 3′-untranslated regions (3′-UTRs) of a target mRNA for subsequent repression or degradation [36–38]. Studies have uncovered the expression profiles and regulatory roles of miRNAs during embryogenesis and early life development. Comparative analysis revealed dynamic changes in miRNAs and their targets during embryonic stem cell (ESC) maintenance and differentiation process. Notably, miRNAs were secreted and transferred into the uterine fluid, whose contents were proposed to be involved in a crosstalk between the mother and conceptus. The maternal nutritional environment undoubtedly affected the utero status and the miRNAs of either maternal or embryo origin, impacting the development of the embryo [39].

## 4. Metabolites Play a Role in Epigenetic Mechanism

Stem cell fate determination is affected by changes in transcriptional programs, which lead to a defined cell lineage under certain microenvironment stimuli [40]. The important role of epigenetics in driving stem cell fate has been widely investigated at and between different regulatory levels such as chromosomal, transcriptional, and posttranscriptional levels [41–43]. Recent studies reported evidence that the regulation of epigenetics not only affects the chemical modification of DNA and histones but also is closely linked with the nutritional status [44]. An essential role of nutrition and nutrition-related metabolism is generating amino acids and other metabolites in rapidly dividing cells [45]. Furthermore, the metabolite levels in stem cells have a direct influence on the epigenome through histone and DNA modifications and expression of miRNAs [46–48].

Generally, nutrition and micronutrients involved in metabolic pathways can interfere with epigenetic mechanism in different ways: the utilization of the methyl groups from OCM in the (1) DNA methylation and (2) histone modification by shifting the activity of methyl transferase. (3) The metabolic status alters miRNA profiles, and in turn, the OCM-related genes could be regulated by miRNA [49, 50]. For these above reasons, micronutrients and metabolic status, influenced by dietary habits, play an essential role in regulating epigenetic modification and stem cell determination during the early stage of fetal development.

### 4.1. OCM and DNA Methylation

During embryonic development, epigenetic reprograming occurs with changes in DNA methylation patterns [27]. Evidence indicates that the process of DNA methylation is susceptible to nutritional status and OCM-related micronutrients including methionine, folate, vitamin B_12_, and vitamin B_6_ [51–55].

In humans, micronutrients from diet influence the production of the methyl groups from OCM and subsequently affect the methylation of DNA [21, 56]. Different feeding strategies of female larvae were found to result in two different phenotypes in honeybees. Barchuk et al. [57] found a total of 240 differentially expressed genes that were activated in early larval stages stimulated by different nutrition status. DNA methylation, influenced by the nutritional input, further impacted the honeybee's developmental fate [58]. Among OCM-related micronutrients, methionine is vital for epigenetic reactions to methylate cytosine in CpG islands. High dietary supplementation of methionine would alter mammalian OCM and increase the DNA methylation status, thus potentially regulating the expression of epigenetically labile genes [59]. In the folate cycle of OCM, folate is catabolized to a series of metabolites that serve as the methyl group donors, which feed into the methylation cycle and convert Hcy to methionine (Figure 1). Upon feeding murine offspring with low-folate diet, epigenetic marks were observed to persist into adulthood [60]. Some studies reported that the maternal folate intake can influence the methyl pool in folate-mediated OCM and the patterns of DNA methylation in the placenta [61]. Additionally, other B vitamins also act as cofactors to support methylation reactions [21]. Maternal vitamin B_12_ level in serum was inversely correlated with the global methylation status of offspring at birth [62]. Maternal choline and betaine intake have potential effects against the methylation process in male infants' cord blood [63].

Nutrition can affect the utilization of the methyl groups by shifting the activity of methyltransferases catalyzing the methylation cycle [12]. SAM and SAH levels could indicate transmethylation potential and methylation status to a certain extent. SAM is converted into SAH by DNMT; conversely, a high SAH concentration inhibits the DNMT activity [64]. As described by Yi et al. [65], high affinity of cellular methyltransferases to SAH results in reduced methylation reactions. It was suggested that the deficiency of folate cycle might increase SAH levels and thereby negatively affect the cellular methylation reactions. In addition, glycine N-methyltransferase (GNMT) also regulates the ratio of SAM/SAH in the methylation cycle [66], and its enzymatic activity was further found to be inhibited by the 5-methyl-THF in folate cycle [67, 68].

Thus, transmethylation metabolic pathway is closely related to the methionine and folate-related cycles, which in turn are associated with several micronutrients. If these micronutrient levels are altered, these pathways may cause compensatory changes that influence the DNA methylation status [59, 69]. It was revealed that the dynamic DNA methylation patterns throughout the life period are regulated by OCM process [70, 71].

### 4.2. OCM and Histone Modification

Methyl deficiency can also influence the regulation of histone modifications by the OCM pathway. The effects of a methyl-deficient diet on histone methylation patterns were found to be similar to that caused by the alternation of DNA methylation resulting in deficiency of the methyl groups [72–74]. Various studies identified that lack of nutrients like methionine, choline, folic acid, and vitamin B_12_ causes aberrant SAM content and impacts the histone modification profiles; as a result, associated epigenomic changes influence the cell activity and lineage fate [75, 76].

The metabolome could regulate epigenetic modifications from preimplantation to postimplantation during embryonic stem cell transition in the early life development. In mouse ESCs, the histone methylation marks can be regulated by threonine deficiency leading to decreased accumulation of SAM [77]. In another study with human ESCs, the depletion of methionine was found to decrease SAM levels, leading to a decrease in H3K4me3 marks and defects in cellular self-renewal [47]. These two studies indicate the crucial role of SAM in regulating ESC differentiation. Mechanistically, these studies focused on threonine and SAM metabolism associated with energy production and acetyl-coA metabolism. The term “methylation index” was used to describe the ratio of SAM to SAH; the influence of SAM/SAH in embryonic stem cells is important part of the interaction between micronutrient and epigenetics. Further studies identified that aberrant SAM/SAH status caused by different levels of methyl diet directly affected histone modifications. Zhou et al. [78] reported that an imbalanced methyl diet resulted in a decrease in SAM level and an upregulation of histone lysine methyltransferase- (KMT-) 8 level in the livers of mice. However, a methyl-deficient diet caused a decrease in histone H3K9me3, H3K9ac, and H4K20me3 in hepatic tissues [74], as a result of which the cell cycle arrest was released. In intestinal stem cells, deprivation of methionine also resulted in cell proliferation and promoted lineage differentiation [79]. Furthermore, Mentch et al. [80] revealed that methionine metabolism plays a key role in regulating SAM and SAH. This dynamic interplay causes changes in H3K4me3, resulting in altered gene transcription as a feedback to regulate OCM. Certain amounts of methionine were required in the maintenance of hESCs and induced pluripotent stem cells (iPSCs). Methionine deficiency resulted in reduced intracellular SAM and NANOG expression by triggering the p53-p38 signaling pathway, potentiating the differentiation of hESCs and iPSCs into all three germ layers. Notably, a prolonged period of methionine deficiency resulted in cellular apoptosis [47]. These findings suggest that SAM status in OCM plays a key role in maintaining stem cells in an undifferentiated pluripotent status and in regulating their differentiation process. Additionally, the nuclear lysine-specific demethylase 1 (LSD1), a histone demethylase, was identified to be a folate-binding protein with high affinity [81]. It was suggested that folic acid participates in the demethylation of histones and thereby functions in regulating gene expression. However, its relationship with OCM needs to be further investigated.

### 4.3. OCM and miRNA

In mice fed with a methyl-deficient diet, a total of 74 miRNAs were differentially expressed in the liver, suggesting a relationship between the expression of miRNAs and methyl deficiency [82]. To further study the potential ability of miRNA in regulating OCM, a computational Monte Carlo algorithm was used to identify candidate master miRNAs of 42 OCM-related genes. As a result, miR-22 was identified as a novel and top OCM regulator that targeted OCM genes (MAT2A, MTHFR, MTHFD2, SLC19A1, TCblR, and TCN2) involved in the transportation, distribution, and methylation of folate and vitamin B_12_. The results also suggested that miR-344-5p/484 and miR-488 function cooperatively as master regulators of the OCM cycle [49]. Using DNA sequencing and by establishing gene network, a total of 48 genes involved in the folate-related OCM pathway were extracted from the KEGG pathway and literature survey. Using this information, a complex database was generated including CpGs, miRNAs, copy number variations (CNVs), and single-nucleotide polymorphisms (SNPs) underlying the OCM pathways (http://slsdb.manipal.edu/ocm/) [83]. Based on these data, recent studies have focused on the potential mechanism between OCM and miRNAs. Song et al. [84] found that the folate exposure of chondrocytes, obtained from individual with osteoarthritis (OA), caused an increase in levels of hydroxymethyltransferase- (HMT-) 2, methyl-CpG-binding protein- (MECP-) 2, and DNMT-3B. Additionally, they reported that miR-373 and miR-370 may, respectively, target MECP-2 and SHMT-2 to directly regulate OCM. Koturbash et al. [85] and Koufaris et al. [86] demonstrated the inhibitory role of miR-29b and miR-22 in regulating the expression of OCM-related genes, including methionine adenosyltransferase I, alpha (Mat1a), and 5,10-methylenetetrahydrofolate reductase (MTHFR). These investigations also showed the role of miR-22 as a regulator in stem cell differentiation and cancer development.

In recent years, the bidirectional analysis of the interplay between miRNA profiles and folate status was examined and the strong interaction between OCM and miRNA expression was shown [87]. In folate-deficient media, cultured mESCs showed differential expression of 12 miRNAs and failed to proliferate and underwent apoptosis. In particular, miR-302a was found to mediate these effects of folate by directly targeting the Lats2 gene [88]. Furthermore, maternal folate supplementation during the late stage of development could restore the folate deficiency-associated defects such as the cerebral layer atrophy and interhemispheric suture defects [89, 90]. These findings suggest that folate deficiency-associatedconsequences might be mediated by miRNAs, indicating their critical roles in mammalian development. Though multiple lines of evidence clearly show the role of miRNAs in regulating OCM and OCM-related genes, there is still a need to elucidate the direct mechanism between nutritional status and functional miRNAs and the potential role of these miRNA as prognostic factors for diseases.

## 5. Future of Dietary Epigenetic Modulators

Since nearly a century, researchers have identified embryonic cells with stable but epigenetically distinct states of pluripotency [91, 92]. Maternal environment and nutrient status can influence the metabolism of fetus through epigenetic modifications in early stage of fetal development. OCM is a crucial metabolic process involving methyl transfers from micronutrients in a cyclical process. The donation and transfer of the methyl groups link the nutrient status to epigenetic mechanism involved in modulation of cellular activities during early development. Notably, epigenetic mechanisms can also modify metabolism and influence the signaling cascades involved in metabolic regulation [93].

In summary, epigenetic factors and metabolic mechanisms form a complex network regulating the cell fate determination during developmental processes. Detailed investigation on the potential mechanism underlying the effect of maternal dietary factors on epigenome modulations of offspring is needed. Furthermore, improvement of dietary component for achieving favorable effects on the epigenetic pattern of the organism may be a promising therapeutic strategy that should be explored.
